# Pregnancy After Bariatric Surgery: Hepatobiliary Implications, Maternal Outcomes, and Clinical Considerations

**DOI:** 10.7759/cureus.98939

**Published:** 2025-12-10

**Authors:** Su Zarni, Min Zin Oo, Thiri Wai

**Affiliations:** 1 Obstetrics and Gynaecology, Luton and Dunstable University Hospital, Luton, GBR; 2 Surgery, University Hospitals Bristol and Weston NHS Foundation Trust, Weston-Super-Mare, GBR; 3 Obstetrics and Gynaecology, University of Medicine 1, Yangon, Yangon, MMR

**Keywords:** bariatric surgery, gastric banding, gastric sleeve, hepatobiliary, obesity, pregnancy

## Abstract

The global rise in bariatric surgery among women of reproductive age has led to an increasing number of pregnancies occurring after significant metabolic and anatomical changes. While bariatric surgery improves fertility and metabolic health, its long-term impact on hepatobiliary physiology during pregnancy remains incompletely understood. This narrative review explores the hepatobiliary implications of pregnancy following bariatric surgery, summarizes maternal and fetal outcomes, and highlights clinical considerations for multidisciplinary management. A comprehensive search of PubMed, Scopus, and Web of Science databases was conducted for studies published between 2000 and 2025, using the terms “bariatric surgery,” “pregnancy,” “liver function,” “gallstones,” “cholestasis,” and “maternal outcomes.” Relevant clinical studies, reviews, and case reports were analyzed and synthesized narratively. Animal studies, non-English-language articles, and studies without pregnancy or hepatobiliary outcomes were excluded from analysis. Pregnancy after bariatric surgery is associated with improved metabolic and obstetric profiles compared with pregnancies in untreated obesity. However, rapid weight loss and altered bile acid metabolism predispose patients to hepatobiliary complications, including gallstone formation, biliary colic, and nutritional liver dysfunction. Liver function test abnormalities are frequent but often transient. Early conception (<12 months post-surgery) increases the risk of micronutrient deficiencies and hepatocellular stress. Close monitoring, nutritional optimization, and coordinated care among obstetricians, surgeons, and hepatologists are essential. Pregnancy following bariatric surgery presents unique hepatobiliary challenges requiring individualized, multidisciplinary management. Further research is needed to elucidate the pathophysiologic mechanisms linking altered bile acid metabolism and hepatic adaptation in this population.

## Introduction and background

The global rise in obesity has led to an increasing number of women of reproductive age undergoing bariatric surgery, now recognized as one of the most effective treatments for long-term weight loss and metabolic improvement [1]. Between 1993 and 2016, an estimated 1,903,273 bariatric procedures were performed in the United States. The mean patient age was 43.9 years, and most were women (79.9%), White (70.9%), and had commercial insurance (70.7%). Over this 23-year period, the demographic and socioeconomic characteristics of bariatric surgery patients changed, reflecting wider access and evolving clinical practice. These data show that bariatric surgery is most often performed in women of reproductive age, consistent with global trends and highlighting the increasing importance of managing pregnancies after surgery [2].

Bariatric surgery improves metabolic health and helps restore fertility by enhancing ovulatory function and insulin sensitivity, especially in women with obesity-related anovulation or polycystic ovary syndrome (PCOS) [3,4]. Pregnancies following bariatric surgery are generally associated with lower risks of gestational diabetes, pre-eclampsia, and fetal macrosomia compared with pregnancies in women with untreated obesity [5].

However, these pregnancies can present specific physiological challenges. Rapid weight loss after surgery, changes in gastrointestinal anatomy, and altered bile acid metabolism can increase the risk of hepatobiliary problems such as gallstones, biliary colic, and temporary liver function abnormalities [6,7]. These issues are particularly relevant during pregnancy, when hormonal and metabolic demands on the liver are naturally increased [8].

Despite growing clinical experience, the liver and biliary effects of pregnancy after bariatric surgery are still not well understood. Evidence remains limited, with few studies exploring how changes in bile acid metabolism, nutrition, and liver function interact during pregnancy [9]. This review aims to (1) examine hepatobiliary changes in pregnancy after bariatric surgery, (2) summarize maternal and fetal outcomes, and (3) highlight key clinical considerations.

## Review

Overview of bariatric surgery and postoperative physiology

Types of Bariatric Procedures

The four major bariatric procedures commonly performed are sleeve gastrectomy (SG), Roux-en-Y gastric bypass (RYGB), adjustable gastric banding (AGB), and biliopancreatic diversion (BPD) with or without duodenal switch (DS) [10]. Most of these surgeries are now conducted laparoscopically, as minimally invasive techniques are associated with lower risks of infection, hernia, and postoperative complications compared to open surgery [11]. The procedure should be selected through a multidisciplinary approach, involving surgeons, dietitians, mental health professionals, and internists, and guided by individual patient factors such as body mass index (BMI), comorbidities, metabolic profile, and treatment goals [12,13].

Comparative evidence suggests that BPD/DS provides the greatest weight loss and metabolic improvement but carries higher risks of nutritional deficiencies and adverse events [14]. RYGB and SG offer a favorable balance between efficacy and safety, improving quality of life and yielding significant remission of type 2 diabetes and dyslipidemia [15-17]. AGB, while less invasive, achieves the least weight loss and metabolic benefit [18]. No single operation is optimal for all patients; rather, the choice must weigh the benefits of metabolic improvement against potential nutritional, surgical, and hepatobiliary complications, particularly in women of reproductive age who may conceive after surgery.

Metabolic and Nutritional Consequences

Bariatric surgery leads to substantial weight loss and improvement in metabolic comorbidities, yet it also carries significant risks of metabolic and nutritional complications. Some of the review highlights that the extensive anatomical alterations, especially after malabsorptive procedures such as RYGB and BPD, can result in macronutrient deficiencies (e.g., protein-energy malnutrition) and the most common micronutrient deficits, including vitamin B12, iron, calcium, and vitamin D [19-21]. The risk is higher with more malabsorptive operations, where fat-soluble vitamin loss, trace-element deficiencies, and protein loss are more prevalent. Inadequate intake, altered absorption, and poor adherence to supplementation contribute to these outcomes [21,22]. In addition, post-surgery metabolic consequences such as dumping syndrome, reactive hypoglycemia, bone mineral loss, and electrolyte abnormalities further complicate the nutritional picture [22]. These consequences emphasize the need for life-long nutritional monitoring, tailored supplementation, and proactive multidisciplinary care in patients who have undergone bariatric procedures.

Effects on Reproductive Physiology

Bariatric surgery has profound effects on reproductive physiology, particularly in women of reproductive age with obesity-related dysfunction. Substantial weight loss following surgery often leads to restoration of ovulatory function and menstrual regularity. In a meta-analysis, irregular menstrual cycles and infertility were significantly reduced post-surgery, while miscarriage and congenital malformation rates remained unchanged [23,24]. Improvements are mediated by favorable hormonal shifts: levels of androgens (e.g., testosterone, androstenedione) fall, while sex hormone-binding globulin rises, and follicle-stimulating hormone and luteinizing hormone may increase, supporting improved hypothalamic-pituitary-ovarian axis function [25]. For example, in women with PCOS, bariatric surgery has been shown to reduce hyperandrogenism, improve ovulation, and increase spontaneous conception rates. However, there are nuances: while ovarian hormone profiles improve, some data suggest decreases in anti-Müllerian hormone levels after surgery, raising questions about long-term ovarian reserve [26]. In addition, nutritional and micronutrient deficiencies post-surgery can adversely impact reproductive health and conception outcomes. Collectively, these findings underscore the need for multidisciplinary pre-conception counseling and close monitoring of reproductive function post-bariatric surgery.

Hepatobiliary adaptations in normal pregnancy

Physiologic Liver Changes

Pregnancy induces extensive physiological adaptations to support maternal-fetal health, including hormonal, circulatory, immunological, and hepatic changes. Elevated estrogen and progesterone levels lead to peripheral vasodilation, reduced vascular resistance, and increased heart rate and stroke volume, resulting in a 40% rise in cardiac output and a 50% increase in plasma volume by the third trimester [27]. Portal venous flow to the liver also increases [28]. Hormonal shifts modulate the immune system, promoting a Th2 anti-inflammatory state and expanding maternal regulatory T cells to protect the fetus [29]. Coagulation factors rise while anticoagulants and fibrinolysis decrease, creating a prothrombotic state [30]. Pregnancy-related hormones influence hepatic metabolism, including cytochrome P450 enzyme activity, bile salt transporter function, and gallbladder motility, predisposing to mild cholestasis and gallstone formation [31]. Liver size enlarges during gestation and lactation, returning to baseline after weaning, and preliminary animal studies suggest pregnancy may enhance hepatic regenerative capacity in older livers [32]. Collectively, these adaptations optimize maternal-fetal exchange but may unmask or exacerbate underlying liver disease.

Pregnancy-Specific Hepatic Disorders

Pregnancy-specific hepatic disorders are unique to gestation and can significantly affect both maternal and fetal outcomes. The major pregnancy-related liver disorders include hyperemesis gravidarum, intrahepatic cholestasis of pregnancy (ICP), preeclampsia with or without HELLP syndrome (hemolysis, elevated liver enzymes, low platelets), and acute fatty liver of pregnancy (AFLP) [33]. These conditions differ in etiology and timing but share overlapping hepatic manifestations such as elevated liver enzymes and jaundice. ICP results from impaired bile acid transport under hormonal influence, whereas HELLP and AFLP involve endothelial dysfunction and hepatic microvesicular steatosis. Early recognition, supportive management, and timely delivery remain the cornerstones of treatment to prevent severe maternal and perinatal complications [34-36].

Relevance to Post-bariatric Context

Pregnancy following bariatric surgery presents overlapping metabolic and hepatobiliary challenges with pregnancy-specific liver disorders. Rapid weight loss and altered bile acid metabolism after bariatric procedures may predispose to cholestasis, steatosis, and gallstone formation, conditions that resemble intrahepatic cholestasis of pregnancy and AFLP [33]. Nutrient deficiencies, particularly in vitamins A, D, E, K, and trace elements, can exacerbate hepatic dysfunction and impair bile acid homeostasis [37]. Both states involve hormonal modulation of hepatic enzymes and oxidative stress, amplifying vulnerability to hepatocellular injury. Understanding this overlap is essential for multidisciplinary monitoring and early detection of hepatic complications in post-bariatric pregnancies.

Hepatobiliary implications of bariatric surgery during pregnancy

In women who become pregnant following bariatric surgery, alterations in anatomy and physiology (e.g., rapid weight loss, nutrient malabsorption, altered bile flows) may heighten hepatobiliary risks, including gallstones, bile duct complications, and liver function test abnormalities. Close monitoring and multidisciplinary care are warranted [38-40].

Gallstone Disease

Rapid weight loss after bariatric procedures promotes bile supersaturation, reduced gallbladder motility, and formation of cholesterol stones. In pregnancy, additional hormonal and metabolic changes amplify risk. Incidence in bariatric cohorts may exceed 30% within the first year [41].

Cholestasis and Bile Acid Dysregulation

Pregnancy-specific cholestasis (e.g., ICP) is marked by impaired bile flow and elevated bile acids. Bariatric surgery may further disrupt enterohepatic bile acid circulation and gut-liver signaling, compounding the risk [42-44].

Liver Function Abnormalities

Mild abnormal liver enzyme results are common in pregnancy, but in the post-bariatric context, altered metabolism, malnutrition, and biliary complications may lead to more pronounced dysfunction. Diagnostic vigilance is required [45].

Nutritional Hepatopathy

After bariatric surgery, micronutrient and macronutrient deficiencies (e.g., vitamins A, D, E, K, protein malnutrition) may affect the liver via fatty change, oxidative stress, or impaired synthetic function, especially when pregnancy adds further nutrient demand [37,40].

Hepatic Adaptation and Regeneration

The maternal liver enlarges and undergoes hepatocyte proliferation during pregnancy, then involutes post-weaning. This dynamic adaptation may be altered following bariatric surgery and influence regeneration or repair responses [32].

Maternal outcomes

Post-bariatric pregnancies generally show lower gestational weight gain and reduced risks of gestational diabetes and hypertensive disorders compared with pre-surgery obesity cohorts. However, some studies report higher odds of small for gestational age (SGA) or preterm birth, underscoring the need for nutrition surveillance and fetal growth monitoring. Evidence varies by procedure and population; multidisciplinary antenatal care is recommended [46,47].

Metabolic and Obstetric Outcomes

Metabolic bariatric surgery improves insulin sensitivity and lowers gestational diabetes and pre-eclampsia rates, and decreases large for gestational age/macrosomia. Balanced against this are increased SGA risk and possible changes in glucose testing (dumping/altered oral glucose tolerance test (OGTT)), requiring tailored screening. Cesarean and induction rates vary by cohort. Nutrient deficiencies (iron, B12, fat-soluble vitamins) should be anticipated and supplemented [48,49].

Hepatic Complications

Most women do well, but clinicians should monitor for liver issues, including ICP, abnormal transaminases from biliary disease or malnutrition, and very rare post-bariatric liver failure (reported after bypass variants). Prompt evaluation of abdominal pain, pruritus, jaundice, or deranged liver function tests is essential. It is important to coordinate with hepatology when indicated [50,51].

Timing of Conception

Guidelines advise delaying conception until weight stabilizes, typically 12-18 (up to 24) months post-surgery, to minimize catabolic state, optimize nutrition, and reduce adverse perinatal outcomes [52]. Consensus statements emphasize preconception counseling, contraception, and supplementation [53]. Emerging data question strict cut-offs but still support individualized planning with careful monitoring at <12-month intervals [54].

Fetal and neonatal outcomes

Offspring of women after bariatric surgery tend to have lower birthweight and higher rates of SGA, exact rates vary, and preterm delivery compared to non-surgery groups, despite reduced large for gestational age incidence [55,56].

Growth and Nutritional Status

Post-bariatric infants may face constrained fetal growth due to maternal nutritional alterations. Studies have shown higher SGA and lower average birth weights, raising concerns about neonatal nutrient stores and early growth trajectories [55,57].

Neonatal Hepatic Function

Data are limited on direct neonatal liver injury after maternal bariatric surgery, but maternal obesity models suggest offspring hepatic fat accumulation and altered enzyme levels. Extrapolation warrants monitoring of neonatal liver markers in this cohort [58].

Long-Term Offspring Effects

Children born after maternal bariatric surgery may carry altered metabolic set-points. Observational studies have linked maternal surgical history with lower offspring obesity risk, yet long-term hepatic and metabolic risks require further investigation [59,60].

Clinical considerations and management

Preconception Counseling

Women who have undergone bariatric surgery should receive targeted pre-conception counseling, including contraception advice, timing of pregnancy (typically 12-24 months after surgery until weight stabilizes), and baseline assessment of nutritional status (iron, B12, folate, vitamin D) and comorbidities. Shared planning with the bariatric, obstetric, and nutrition teams is essential [61].

Antenatal Monitoring

Antenatal care for post-bariatric surgery pregnancies requires enhanced monitoring: serial ultrasound growth assessment (due to elevated SGA risk), frequent nutritional laboratory testing each trimester, adjusted gestational diabetes screening (OGTT may be unreliable in bypass patients), and prompt investigation of any gastrointestinal symptoms (risk of internal hernia/band complications) [62].

Multidisciplinary Approach

Optimal management involves a multidisciplinary team, including obstetricians (preferably maternal-fetal medicine), bariatric surgeons or specialists, dietitians with bariatric expertise, and endocrinologists. This collaboration ensures coordinated care addressing surgical anatomy, nutritional supplementation, metabolic issues, and obstetric planning across the periconception, antenatal, and postpartum phases [48,53].

Postpartum and Long-Term Follow-Up

After delivery, women who underwent bariatric surgery require continued follow-up for nutritional status (ongoing needs in iron, B12, vitamin D, protein), weight trajectories, and metabolic health (e.g., diabetes relapse). Breastfeeding should be supported with attention to maternal nutrient stores. Offspring growth and developmental milestones should also be monitored long-term, given potential early nutritional influences [12,53].

## Conclusions

Bariatric surgery before pregnancy offers significant metabolic and obstetric benefits, including reductions in gestational diabetes, hypertensive disorders, and macrosomia. However, it also introduces complex hepatobiliary and nutritional challenges that demand careful management. Altered bile acid metabolism, gallstone formation, and potential liver function abnormalities can arise due to rapid weight loss and malabsorption. During pregnancy, these physiological changes interact with hepatic adaptation, occasionally leading to cholestasis or transient enzyme derangements. Preconception counseling and individualized timing of conception, preferably 12-18 months post-surgery, remain critical for optimizing maternal and fetal outcomes. Antenatal care should include trimester-based nutritional screening, growth monitoring, and vigilance for hepatic or surgical complications. A multidisciplinary team, comprising obstetricians, bariatric specialists, hepatologists, and dietitians, ensures holistic management from conception through the postpartum period. Although most post-bariatric pregnancies proceed successfully, emerging evidence highlights the need for long-term follow-up to assess metabolic, hepatic, and developmental outcomes in both the mother and the child. Future research should focus on mechanistic links between altered bile acid homeostasis, hepatic regeneration, and fetal programming. In conclusion, bariatric surgery represents both a therapeutic opportunity and a clinical challenge in pregnancy, necessitating coordinated, evidence-based care to safeguard maternal hepatic health and promote optimal neonatal growth.
